# Mechanism of physicochemical quality formation in *Polygonatum odoratum* powder prepared by vacuum microwave drying combined with extrusion-cooking: a metabolomics-based analysis

**DOI:** 10.3389/fnut.2026.1950920

**Published:** 2026-09-16

**Authors:** Fan Jia, Yao Xu, Yuhang Wu, Jiani Jiang, Tao Zheng, Hui Zheng, Yong Yang

**Affiliations:** College of Pharmacy, Hunan University of Chinese Medicine, Changsha, China

**Keywords:** antioxidant activity, metabolomics, physicochemical quality, *Polygonatum odoratum*, vacuum microwave drying combined with extrusion-cooking

## Abstract

**Introduction:**

*Polygonatum odoratum* (*P. odoratum*) is the dried rhizome of plant *Polygonatum odoratum* (Mill.) Druce, and it is a well-known medicinal and edible plant extensively utilized in Asia, but its commercial products, sliced or strip forms, limiting its wider applications. The powdered form of *P. odoratum* offers greater convenience for modern food applications. This study aims to develop efficient processing strategies for high-quality *P. odoratum* powder.

**Methods:**

This study used fresh *P. odoratum* as raw material to investigate the quality of powder produced by vacuum microwave drying combined with extrusion cooking (combined approach) and compared it with powders obtained by hot-air drying and vacuum microwave drying alone. Electronic sensory technology, including electronic eye (e-eye), electronic noses (e-nose), and electronic tongues (e-tongue), were utilized to evaluate physical properties, *in vitro* antioxidant activity assays were used to preliminarily evaluate functional activity, and chemical analytical methods were employed for analyzing the main active components. Meantime, the differences in saponins and flavonoids among different processed products were determined by broad-targeted metabolomics techniques. Finally, correlation analyses of physical properties, antioxidant activity, and chemical properties were performed to investigate the mechanism underlying the formation of product quality.

**Results:**

The results showed that the combined approach enhanced the physicochemical properties and *in vitro* antioxidant activity of *P. odoratum* powder, primarily by effecting its flavonoid metabolites. Specifically, a deeper yellow color was achieved through reduced Phloretin content and increased Eriodictyol chalcone content; sweetness was enhanced by higher Eriodictyol levels; and antioxidant activity was enhanced by increased Eriodictyol content and the emergence of Epigallocatechin gallate.

**Conclusion:**

The *P. odoratum* powder prepared by vacuum microwave drying combined with extrusion cooking process in this study exhibited high quality, and the process showed good industrial feasibility.

## 1 Introduction

*Polygonatum odoratum* (*P. odoratum*), a traditional Chinese medicinal (TCM) material, is the dried rhizome of plant *Polygonatum odoratum* (Mill.) Druce, which belongs to the genus Polygonatum in the family Liliaceae. In TCM, *P. odoratum* is considered to have the health functions of nourishing yin, moistening dryness, generating fluids, and quenching thirst. It has been widely used in TCM clinical practice and dietary therapy for health maintenance in China and Southeast Asia, commonly prepared as decoctions. Modern research has indicated that *P. odoratum* and its extracts, including polar extracts of rhizomes, aqueous extracts and rhizome decoctions, exhibit multiple health effects, such as antioxidant activity (1), hypoglycemic effects (2), antitumor properties (3), immune modulation (4), antibacterial activity (5), hepatoprotection (6), and anticancer effects (7). These pharmacological activities are primarily attributed to its bioactive constituents, including saponins (polygonatoside A–D), flavonoids (polygonatone A–D), alkaloids (polygonatine A and polygonatine B), and polysaccharides serve as the material basis for these health benefits (8, 9).

Although *P. odoratum* possesses diverse pharmacological activities and health benefits, its commercial products are largely confined to traditional sliced or strip forms, thereby limiting their wider applications and reducing their potential value in the development of functional foods and nutraceuticals. Compared with strip or slice forms, the powdered form of *P. odoratum* offers greater convenience for modern food applications. For example, powder processing can significantly broaden the application range of *P. odoratum, P. odoratum* powder exhibits excellent dispersibility and blending compatibility, and powder processing offers advantages in storage, transportation, and preservation. Due to the high polysaccharide content in *P. odoratum*, the traditional powder preparation process (typically involving steaming and drying of fresh *P. odoratum* before grinding) is not only complex but also quite difficult, often leading to undesirable characteristics in the final product, such as poor color, subpar flavor, and loss of functional components. Therefore, exploring new grinding processes is crucial for improving the quality of *P. odoratum* powder and expanding the application range.

Vacuum microwave drying (VMD) is an innovative drying method that combines microwave heating and vacuum evaporation. VMD combines the dual characteristics of microwave-heating materials from inside and vacuum-enhancing moisture evaporation, achieving rapid drying with minimal impact on raw material composition (10). Numerous studies have found that VMD can increase the drying rate and the retention rate of functional components (e.g., total polyphenol, total saponins and total polysaccharides), while also enhancing the functional activity of dried products (11, 12).

Extrusion-cooking, a modern thermal processing technology widely applied in food and feed processing, integrates multiple unit operations, including conveying, mixing, heating, pressurization, and shearing (13–15). Due to the simple and effective advantages of extrusion-cooking technology, its applications in new fields such as functional materials and their composite materials are increasing daily. Extrusion-cooking enhances product palatability, making inherently unpalatable foods more acceptable to consumers. Li et al. (16) have found that extrusion-cooking reduced bitterness and enhanced sweetness in quinoa, effectively improved its acceptability. Extrusion-cooking can significantly alter the structure of raw materials, facilitating the dissolution of functional components from materials, particularly polysaccharides, flavonoids, and saponins. Zhang et al. (17) observed that brown rice processed by extrusion-cooking exhibited a 232.26% increase in free total polyphenols. In addition, after extrusion-cooking, the polysaccharides content of Pinellia tuber (18), Hericium erinaceus (19), and Ganoderma lucidum (20) was significantly increased, and the saponins content of red ginseng and white ginseng (21) was significantly increased. And extrusion-cooking also enhances the pharmacological and health-promoting benefits of certain TCM products, such as the *in vitro* antioxidant capacity of Rhodiola (22).

To explore efficient processing methods for producing high-quality *P. odoratum* powder, this study focused on VMD and extrusion-cooking technologies for powder preparation. Based on fresh grinding, different processing methods, including hot-air drying, VMD, and VMD combined with extrusion-cooking, were used to prepare *P. odoratum* powder. The physicochemical quality of the powders obtained under different conditions was analyzed using electronic sensory analysis, chemical analysis, and metabolomics techniques. Separately, electronic sensory technology, including electronic eye (e-eye), electronic noses (e-nose), and electronic tongues (e-tongue), were utilized to evaluate physical properties, *in vitro* antioxidant activity assays were used to preliminarily evaluate functional activity, and chemical analytical methods were employed for analyzing the main active components, such as total saponins and total flavonoids. Meantime, the differences in saponins and flavonoids among different processed products were determined by broad-spectrum metabolomics techniques. Finally, correlation analyses of physical properties, antioxidant activity, and chemical properties were performed to investigate the mechanism underlying the formation of product quality.

## 2 Materials and methods

### 2.1 Plant material

Fresh *P. odoratum*, free from any special lesions and injuries, were harvested on December 8, 2023, from Tianhong Pharmaceuticals planting base in Shaoyang City, Hunan Province, China (111°52′ E, 27°17′ N; altitude 286 m). To reduce experimental variability, fresh *P. odoratum* samples were collected from a single field, ensuring uniform cultivation conditions and growth environment.

### 2.2 Chemicals

HPLC-grade methanol (CH_3_OH) was purchased from Merck (Darmstadt, Germany). HPLC-grade acetonitrile was purchased from Shanghai Xingke (Shanghai, China). HPLC-grade formic acid was purchased from Aladdin (Shanghai, China). 1,1-diphenyl-2-picryl-hydrazyl radical (DPPH•), 2,2′-Azinobis (ABTS^+^•), 2,4,6-Tri(2-pyridyl)-s-triazine (TPTZ), and Trolox were purchased from Macklin Company (Shanghai, China). Shanghai Yuanye Biotechnology Co., Ltd. (Shanghai, China) provided FeCl3, HCl, ethanol, and other reagents purchased from Sinopharm Chemical Reagent Co.

### 2.3 Sample preparation

*P. odoratum* powder prepared by hot-air drying (HA): Fresh *P. odoratum* was washed, cut into 2–3 cm cubes, and then shaved into thin slices using a bc-386 ice shaver (Rongshi Da, Hefei, China). 2.5 kg of thin slices and 250 mL of purified water were placed into a colloid mill (Zhejiang Haoxing, China) and ground into a homogeneous pulp. The pulp was spread on trays and dried in a hot-air drying oven (model 101-3AB; Beijing Zhongxing Weiye, China) and dried at 65 °C for 18 h to the moisture content below 10%. The dried material was ground and passed through a 60-mesh sieve, and the resulting HA powder was packed in airtight containers.

*P. odoratum* powder prepared by VMD (VM): Prepared fresh *P. odoratum* pulp according to the method described in “HA.” The pulp was spread on trays and dried in a VMD oven (model WB-5; Famo Uco, China) and dried at 65 °C and −0.08 MPa for 5 h to the moisture content below 10%. The dried material was ground and passed through a 60-mesh sieve, and the resulting VM powder was packed in airtight containers.

*P. odoratum* powder prepared by VMD combined with extrusion-cooking (EC): The EC was prepared based on the VM. The VM was fed into an extrusion puffing machine (Shandong Mingben, China) for extrusion-cooking. The parameters of extrusion-cooking were as follows: barrel temperatures of 40 °C (first zone), 60 °C (second zone), and 90 °C (third zone); main screw speed, 15 rpm; feeding speed, 9 rpm. After extrusion-cooking, the material was ground and passed through a 60-mesh sieve, and the resulting EC powder was packed in airtight containers.

### 2.4 Electronic sensory analysis

To more accurately and objectively evaluate the physical properties of each *P. odoratum* powder sample, electronic sensory technologies were used to comprehensively analyze the differences in color, odor, and taste.

#### 2.4.1 E-eye analysis

E-eye analysis was performed using a CS-580 spectrophotometer (Hangzhou Caipu). After the instrument was powered on and allowed to stabilize, it was calibrated, and the lens exposure and focal length were set appropriately. Measurements were then performed using a 5 nm aperture and a D65 illuminant with top and bottom illumination. *P. odoratum* powder samples prepared by different processing methods were evenly spread over the measurement aperture. Each sample was measured three times, and the average value was recorded.

The color of each sample was characterized by the CIE 1976 L^*^a^*^b^*^ system with four indicators: L^*^ (lightness), a^*^ (redness/greenness), b^*^ (yellowness/blueness), and $E_{\text{ab}}^{*}$ (the total colorvalue). The L^*^ value ranges from 0 (black) to 100 (white), positive a^*^ values indicate red and negative a^*^ values indicate green, positive b^*^ values indicate yellow and negative b^*^ values indicate blue. The $E_{\text{ab}}^{*}$ value, serving as an auxiliary index derived from L^*^, a^*^, and b^*^ values, which was computed as an integrated index of the overall color alteration according to Equation 1 (a lower $E_{\text{ab}}^{*}$ indicates a whiter color) and enables quantitative evaluation of color differences between samples.


$$\begin{array}{l} {E^{*}}_{ab}=\sqrt{L^{*2}+a^{*2}+b^{*2}} \end{array}$$ (1)

#### 2.4.2 E-nose analysis

Preparation of the test solution: A precisely weighed 1 g of *P. odoratum* powder was placed into a 20 mL headspace vial, capped, and allowed to equilibrate at room temperature for 2 h to detection. E-nose analysis was performed using a PEN3 e-nose (Airsense, France). The measurement parameters were as follows: injection volume 5,000 μL, injection speed 125 μL/s, purge time 120 s, backfill speed 500 μL/s, inlet temperature 200 °C, inlet pressure 10 kPa, outlet flow rate 30 mL/min, injection time 45 s, incubation at 40 °C for 300 s, valve temperature 250 °C, detector temperature 260 °C, and trap temperature 50 °C.

#### 2.4.3 E-tongue analysis

Preparation of the test solution: A precisely weighed 3 g of *P. odoratum* powder was soaked in 100 mL of distilled water for 30 min. Then the mixture was sonicated at 30 °C for 45 min and centrifuged at 4,000 r/min for 10 min. The resulting supernatant was transferred to a 100 mL volumetric flask and diluted to the mark with purified water to obtain the test solution for e-tongue analysis.

E-tongue analysis was performed using an SA402B e-tongue (Insent, Japan). After all sensors were activated and self-tested according to the manufacturer's instructions, sample testing was initiated. The measurement parameters were as follows: alternating acquisition and cleaning cycles, measurement time 90 s, acquisition interval 1 s, acquisition delay 0 s, cleaning time 360 s. Each sample was tested in quadruplicate, and the average of the last three measurements was calculated as the final result. However, sweetness was measured five times, and the most stable data from the last three measurements was calculated as the final result.

### 2.5 *In vitro* antioxidant activity assay

Preparation of the test solution: A precisely weighed 5 g of *P. odoratum* powder was soaked in 50 mL of 70% methanol, the mixture was sonicated at 25 °C for 30 min and centrifuged at centrifuged at 8000 r/min for 10 min. The resulting supernatant was the test solution.

DPPH• and ABTS^+^• scavenging rates and ferric iron reducing antioxidant power (FRAP) were determined according to the method described by Zheng et al. (23).

The determination method for DPPH• scavenging rate was determined as follows: 0.2 mL of the test solution was mixed with 3.8 mL of DPPH• solution, the mixture was placed in the dark at 25 °C for 30 min, and the absorbance was measured at 515 nm.

The determination method for ABTS^+^• scavenging rate was determined as follows: 0.2 mL of the test solution was mixed with 3.8 mL of ABTS^+^• working solution (prepared with an absorbance value of 0.70 ± 0.02 at 734 nm). The mixture was placed in the dark at 25 °C for 7 min, and the absorbance was measured at 734 nm.

The determination method for FRAP was determined as follows: 0.1 mL of the test solution was mixed with 4.9 mL of the FRAP reagent, the mixture was placed at 37 °C for 10 min, and the absorbance was measured at 593 nm. The FRAP reagent was prepared by mixing 300 mM acetic acid buffer (pH 3.6), 10 mM 2,4,6-tri(2-pyridyl)-1,3,5-triazine (TPTZ), and 20 mM FeCl_3_ (10:1:1).

All absorbance values were measured using a UV-1780 spectrophotometer (Shimadzu, Kyoto, Japan). A Trolox standard curve was prepared and measured under the same conditions as the samples for each assay, and the results were expressed as μmol Trolox equivalents per gram dry basis (μmol Trolox/g). *In vitro* antioxidant activity was expressed as Trolox equivalents (μmol Trolox/g).

### 2.6 Determination of component group content

To explore how VMD combined with extrusion-cooking affects the physical properties and *in vitro* antioxidant activity of *P. odoratum* powder, total flavonoids and total saponins were chosen as quality indicators. In addition to being major contributors to the antioxidant activity, flavonoids impart a yellow color and saponins confer bitterness, both of which are relevant to the physical and sensory properties. These compounds are also readily degraded under the high-temperature and high-shear of extrusion-cooking, making them sensitive markers of process-induced changes. In contrast, total polysaccharide content remained largely unchanged and was therefore not used as an evaluation criterion.

#### 2.6.1 Determination of total saponins content

Total saponins content (TSC) was determined according to the method of Jia et al. (24). Curved line plotting: 40, 80, 120, 160, 200, and 240 μL of 1.0 mg/mL dioscin reference solution was precisely pipetted into colorimetric tubes, respectively. evaporated the solvent at low temperature, added 0.2 mL of 5% vanillin-chilled acetic acid solution, another added 0.8 mL of perchloric acid, shake well, and bath in 60 °C for 20 min, then cool quickly after removal. Finally, 5 mL of glacial acetic acid solution was added, the mixture was shaken well, and it was placed in the dark for 30 min. Subsequently, the absorbance was measured at 560 nm and a curved line was plotted. The curved line was *y* = 0.6444*x* + 0.0037, *R*^2^ = 0.9999.

Test solution preparation: 10 mL of the sample was dilute with water to approximately 25 mL. Subsequently, 20 mL of water-saturated n-butanol was added, shaken, and the *n*-butanol layer solution was separated. This procedure was repeated three times. The *n*-butanol solutions were combined, and 20 mL of ammonia water was added for washing. The ammonia water layer was discarded. This procedure was repeated twice. The n-butanol solution was evaporated to dryness in a water bath, the residue was transferred to a 10 mL volumetric flask, and dissolved with methanol to the mark, and mixed thoroughly.

The method for determining TSC followed the curve line plotting procedure, and the accompanying reagent was used as a blank control to measure the test solution absorbance at 560 nm. TSC was calculated using the curve line and based on dry weight (DW) after deducting the sample moisture content (mg/g DW).

#### 2.6.2 Determination of total flavonoids content

Total flavonoids content (TFC) was determined according to the method of Zheng et al. (25). Curved line plotting: 0, 0.2, 0.4, 0.6, 0.8, 1.0, and 1.2 mL of 0.2 mg/mL rutin reference solution was precisely pipetted into 10 mL colorimetric tubes, respectively. Then, 0.5 mL of 5% NaNO_2_ solution was added, the mixture was shaken well, and it was allowed to stand for 6 min. Then, 0.5 mL of 10% Al(NO_3_)_3_ solution was added, shaken well, and allowed to stand for 6 min. Finally, 4 mL of 4% NaOH solution was added, and the solution was diluted to the tick mark with 40% ethanol, shaken well, and allowed to stand for 10 min. The absorbance was measured at 510 nm, and a curved line was plotted. The curved line was *y* = 0.1996*x* + 0.0017, *R*^2^ = 0.9992.

Test solution preparation is same as part of “*In Vitro* Antioxidant Activity Assay.” Method for determining TFC followed the curve line plotting procedure, and the accompanying reagent was used as a blank control and measured the test solution absorbance at 510 nm. TFC was calculated using the curve line and based on dry weight (DW) after deducting sample moisture content (mg/g DW).

### 2.7 Broad-targeted metabolomics analysis

To further explore the mechanism by which VMD combined with extrusion-cooking affects the physical properties and *in vitro* antioxidant activity of *P. odoratum* powder, a broadly targeted metabolomics analysis was performed to compare the compositional differences among the three samples. The sample were analyzed using an UPLC-ESI-MS/MS system (UPLC, Exion LC™AD; https://sciex.com.cn/) and Tandem mass spectrometry system (https://sciex.com.cn/).

#### 2.7.1 Test solution preparation

50 mg of sample was weighed using the electronic balance (MS105DM). Added 1200 μL 70% methanol internal standard extraction solution pre-chilled to 20 °C. The internal standard extraction solution was prepared by dissolving 1 mg of standard material in 1 mL 70% methanol to obtain a 1,000 μg/mL stock solution. The stock solution (1,000 μg/mL) was further diluted with 70% methanol to prepare a 250 μg/mL internal standard solution. Vortexed every 30 min for 30 s, and totaling six vortexing cycles. Then centrifuged at 12,000 r/min for 3 min, the supernatant was aspirated and filtered through a microporous membrane (0.22 μm pore size), and stored in an injection vial for UPLC-MS/MS analysis.

#### 2.7.2 UPLC conditions

Column: Agilent SB-C18 (1.8 μm, 2.1 mm × 100 mm); Mobile phase: phase A = ultrapure water with 0.1% formic acid, phase B = acetonitrile with 0.1%formic acid; Gradient elution procedure: 0.0–9.0 min, 5%−95% B; 9.0–10.0 min, 95% B; 10.0–11.1 min, 95%−5% B; 11.1–14.0 min, 5% B. Flow velocity: 0.35 mL/min; column temperature: 40 °C; Injection volume: 2 μL. The effluent was alternatively connected to an ESI-triple quadrupole-linear ion trap (Q TRAP)-MS.

#### 2.7.3 ESI-Q TRAP-MS/MS condition

Mass spectrometry conditions: source temperature 500 °C; ion spray voltage (IS): 5,500 V (positive ion mode)/−4,500 V (negative ion mode); ion source gas I (GSI), gas II(GSII) and curtain gas (CUR): 50, 60, and 25 psi, respectively; the collision-activated dissociation (CAD) was high. QQQ scans were acquired as MRM experiments with collision gas (nitrogen) set to medium. DP (declustering potential) and CE (collision energy) for individual MRM transitions was done with further DP and CE optimization. A specific set of MRM transitions were monitored for each period according to the metabolites eluted within this period.

#### 2.7.4 Broad-targeted metabolomics analysis based on LC-MS/MS

Based on the in-house MWDB database (MetWare Biotechnology Co., Ltd., Wuhan, China), compound identification was performed using secondary spectrum information. During analysis, isotope signals, duplicate signals containing K^+^, Na^+^, or ${\text{NH}}_{4}^{+}$, and duplicate fragment ions originating from other higher-molecular-weight compounds were removed. In MRM mode, the quadrupole first selects precursor ions (parent ions) of target compounds, excluding ions from other molecular weight compounds to preliminarily eliminate interference. After collision-induced ionization in the collision cell, precursor ions fragment into numerous fragment ions. These fragment ions are then filtered by the triple quadrupole to select a specific target fragment ion, excluding non-target ion interference for more precise quantification and improved reproducibility. After obtaining metabolite mass spectrometry data from different samples, peak area integration is performed for all substance chromatographic peaks. Mass spectrometry peaks of the same metabolite across different samples undergo integration calibration.

Processed data undergo principal component analysis (PCA) and orthogonal partial least squares discriminant analysis (OPLS-DA), and differential metabolite Venn diagram generation via online software on the MyVee Bioinformatics Cloud Platform. Variable Importance in the Projection (VIP) values are extracted from OPLS-DA results. Differential metabolites are screened based on VIP (VIP > 1) and fold change (FC) criteria (FC > 2). The filtered differential metabolites were annotated and classified using the Kyoto Encyclopedia of Genes and Genomes (KEGG) database (https://www.kegg.jp/kegg/pathway.html). Significantly enriched pathways involving metabolites within a module were compared against the background and defined using hypergeometric tests with a threshold *P* < 0.05.

### 2.8 Statistical analysis

All samples were measured repeatedly under identical experimental conditions. The electronic sensory evaluation was conducted with four independent replicates, whereas the *in vitro* antioxidant activity assays and untargeted metabolomics analyses were performed with three and four independent replicates, respectively. Experimental data are expressed as mean ± standard deviation (*x* ± *s*). For routine quality indicators, one-way analysis of variance (ANOVA) and Tukey's multiple comparison tests were conducted using IBM SPSS Statistics 26 (Almonk, NY, USA). For the Spearman correlation analysis, the correlations between metabolites and color or taste traits were based on 12 paired observations (four replicates × 3 treatment groups), whereas those between metabolites and antioxidant activities were based on 9 paired observations (three replicates × three treatment groups). For metabolomic analysis, differentially abundant metabolites were identified using the Benjamini–Hochberg false discovery rate (FDR) correction method, with an adjusted *P* < 0.05 considered statistically significant. For Spearman correlation analysis between metabolites and quality-related traits, the *P* value of all metabolite–trait combinations were adjusted using the FDR procedure to account for multiple testing. Associations with an adjusted *P* < 0.05 were considered statistically significant. Plotting was performed using Origin (Northampton, MA, USA). *P* < 0.05 indicates statistically significant differences.

## 3 Results and analysis

### 3.1 Electronic sensory characteristics

Electronic sensory technologies, including e-eye, e-nose and e-tongue, are biomimetic detection devices that simulate human vision, olfaction, and taste. These technologies provide more sensitive and objective characterization of sensory properties and enable their digitization. Compared with conventional human sensory evaluation, the application of electronic sensory technologies enhances the accuracy of food quality assessment and is more effective in ensuring product quality (26). Currently, they are becoming increasingly common in the evaluation of food processing, such as for cocoa beans (27), green tea (28), and chrysanthemum (29). Metabolomics has been extensively applied in the food sector due to its outstanding detection and predictive capabilities (30). In this study, e-eye, e-nose, and e-tongue systems were employed to comprehensively evaluate the color, odor, and taste of three types of *P. odoratum* powder.

#### 3.1.1 E-eye results

The e-eye results of three samples are shown in Figures 1A, B, and the actual photographs of those are shown in Figure 1C. As shown in Figure 1A, the b^*^ value of three samples were positive and higher than their a^*^ values, indicating that three types of *P. odoratum* powder exhibited a yellow hue. Compared with HA, the L^*^, b^*^, and a^*^ values of VM were no significant difference, while $E_{ab}^{*}$ of VM is higher than that of HA, indicating that there is difference in color between VM and HA, the color of VM is waiter, which is consistent with the color changes actually observed in practice (Figure 1C). Compared with HA and VM, the $E_{ab}^{*}$ of EC is higher, L^*^ value of EC is lower, a^*^ value and b^*^ are higher, these data indicates that the color of EC is significantly different from that of VM and HA, and the color of *P. odoratum* powder exhibits significant changes after extrusion-cooking. Specifically, the overall color of *P. odoratum* powder after extrusion-cooking becomes darker, more reddish, and more yellowish, which is consistent with the color changes actually observed in practice (Figure 1C).

**Figure 1:**
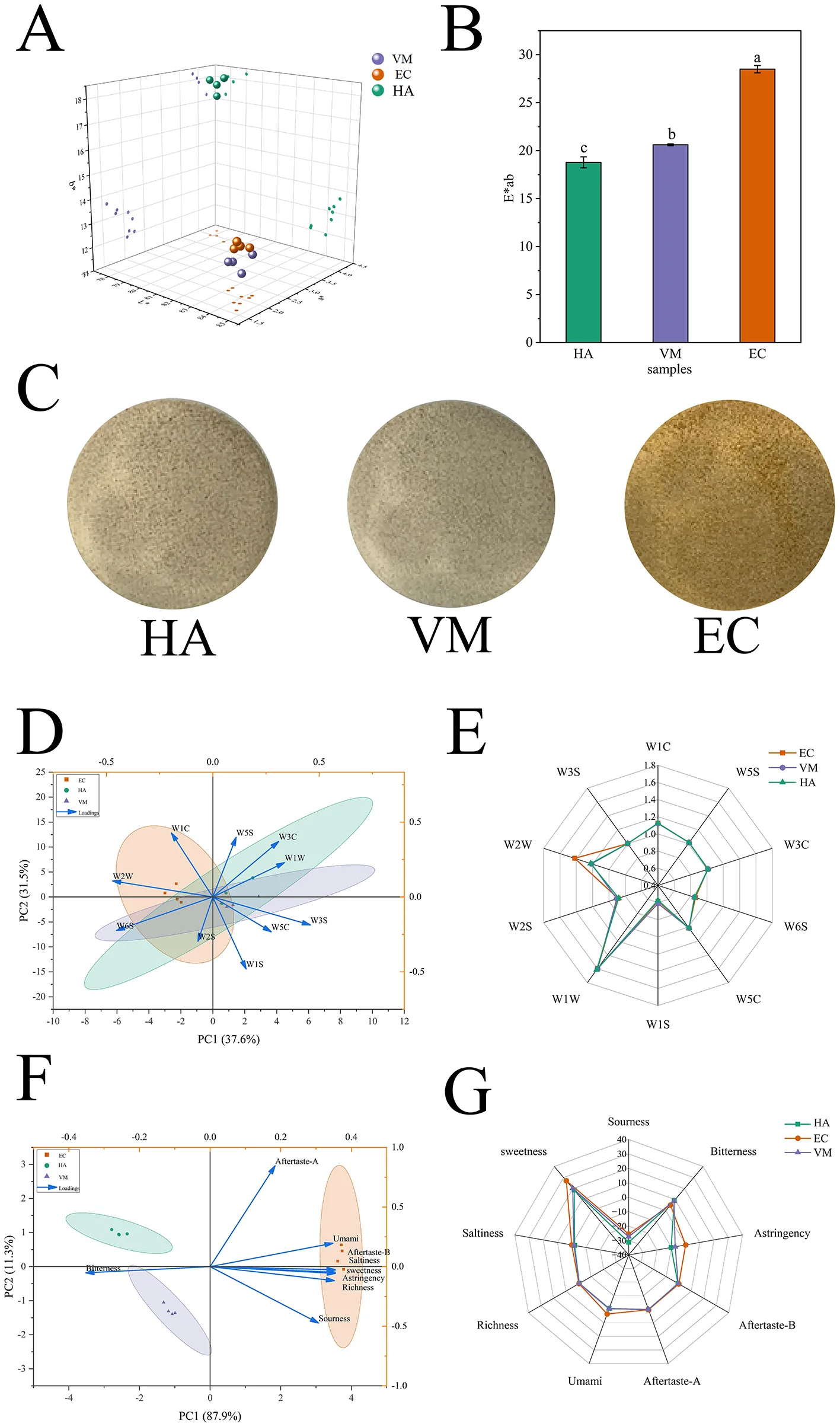
Electronic sensory characteristics of three samples. **(A)** Three-dimensional scatter plot of L*, A*, and b* values. **(B)** $E_{\text{ab}}^{*}$ of three samples. **(C)** Actual photographs of three samples. **(D)** E-nose PCA diagram of analysis for three samples. **(E)** E-nose radar diagram of three samples. **(F)** E-tongue PCA diagram of tea infusions of three samples. **(G)** E-tongue radar diagram of tea infusions of three samples.

#### 3.1.2 E-nose results

The e-nose results of three samples are shown in Figure 1D. Figure 1D is the e-nose PCA diagram of three samples, it shows that three samples are not completely distinct from one another, indicating that different processing methods do not significantly change the odor distribution of *P. odoratum* powder.

Figure 1E presents the e-nose radar plot of the three samples. As shown, the samples differed only in the W2W sensor response, while no significant differences were observed for the other sensors. The W2W sensor is sensitive to aromatic compounds and organosulfur compounds. No significant difference in W2W response was found between HA and VM. In contrast, EC exhibited a significantly higher W2W response than HA and VM, indicating that extrusion-cooking enhanced the aromatic profile of *P. odoratum* powder. One possible explanation for this change is that the Maillard reaction occurred under high temperatures during extrusion-cooking, leading to the formation of new aromatic volatile compounds (31). Another possible explanation is that aromatic compounds previously bound to cell walls were released due to the high shear forces generated during extrusion-cooking (32).

#### 3.1.3 E-tongue results

The e-tongue results of three samples are shown in Figures 1F, G. Figure 1F is the e-tongue PCA diagram of three samples, it shows that the three samples are completely distinct from one another, indicating that different processing methods significantly change the taste characters of *P. odoratum* powder tea infusion.

Figure 1G presents the e-tongue radar plot of the three samples. It illustrates the specific differences in various taste characteristics among the tea infusions of the samples, including sourness, sweetness, saltiness, richness, umami, aftertaste-A (astringency), aftertaste-B (bitterness), astringency, and bitterness. As shown in Figure 1G, compared with HA, the sourness and astringency response values of VM are significantly lower. Compared with VM and HA, bitterness response value of EC is significantly reduced, while sweetness and astringency response values are significantly elevated. These results indicated that extrusion-cooking improves the taste characteristics of tea infusion of *P. odoratum* powder, specifically reducing bitterness and enhancing sweetness.

### 3.2 *In vitro* antioxidant activity

Antioxidant activity is considered one of the key pharmacological effects of *P. odoratum*, serving as a crucial factor for its edible and medicinal applications. However, the impact of different processing methods on the *in vitro* antioxidant activity of *P. odoratum* powder remains unclear. Therefore, the antioxidant capacity of three types of *P. odoratum* powder was evaluated by using DPPH• and ABTS^+^• scavenging assays and FRAP assay, and the results are shown in Figure 2.

**Figure 2:**
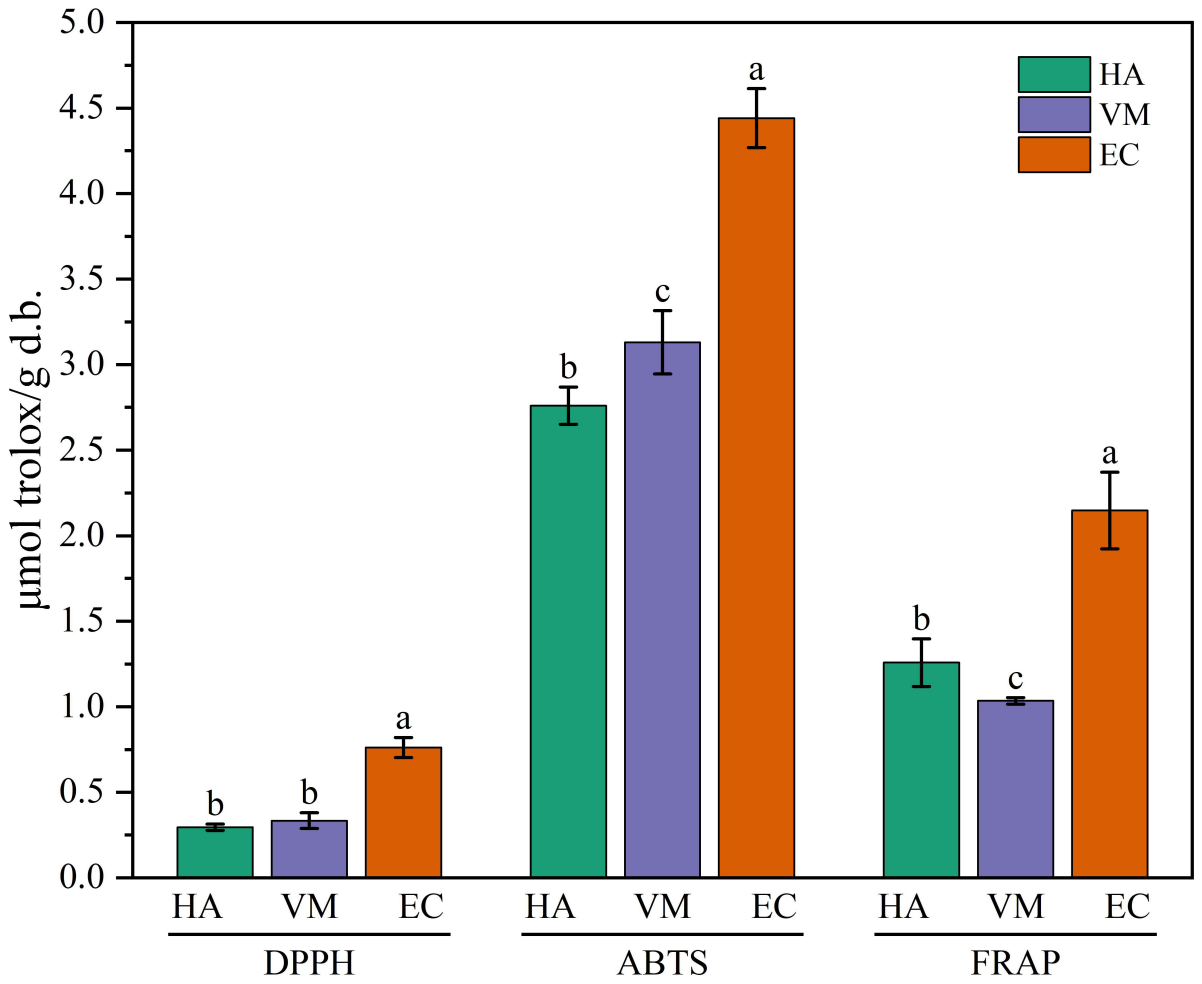
*In vitro* antioxidant activity of three samples. Error bars indicate standard deviation; different lowercase letters denote significant differences (*P* < 0.05).

Figure 2 shows that compared with HA, VM had no significant difference in DPPH• scavenging rates, significantly higher ABTS^+^• scavenging rates (*P* < 0.05), while significantly lower iron-reducing capacity (*P* < 0.05). EC exhibited the highest antioxidant activity across all three assays: DPPH• scavenging rate (0.76 μmol Trolox/g), ABTS^+^• scavenging rate (4.44 μmol Trolox/g), and iron-reducing capacity (2.14 μmol Trolox/g), all significantly higher than those of HA and VM. These results indicate that the *in vitro* antioxidant activity of *P. odoratum* powder was significantly increased after extrusion-cooking, which is consistent with previous findings. For instance, Zhang (22) found that DPPH• scavenging activity, ABTS+• scavenging activity, and total reducing power of Rhodiola rosea were significantly increased after extrusion-cooking processing. Kosińska-Cagnazzo et al. (33) also observed that Lycium complex (rice flour, wheat flour, and Lycium) exhibited a 100% increase in DPPH• scavenging capacity and a 60% increase in ABTS+• radical scavenging capacity after extrusion-cooking processing. This change may be attributed to extrusion-cooking, which promotes the dissolution of antioxidant components within cells, as well as to the Maillard reaction during extrusion cooking, which can stimulate the formation of pigments with antioxidant properties.

### 3.3 Total saponins and total flavonoids content

Preliminary results showed that the extrusion-cooking process improved the physicochemical properties (including color and taste) and *in vitro* antioxidant activity of *P. odoratum* powder, but the mechanisms were unclear. Therefore, the following study compared functional compound contents in three types of *P. odoratum* powder to elucidate the macro-level mechanisms underlying the production of high-quality powder using the VMD combined with extrusion-cooking method.

The total saponins content (TSC) and total flavonoids content (TFC) of the three samples are shown in Figure 3. As illustrated, the TSC and TFC followed the order EC > VM > HA, with EC exhibiting the highest values among the three samples. Specifically, the TSC of EC was 25.21 mg/g DW, which was 1.79 times that of HA and 1.31 times that of VM, respectively. The TFC of EC was 2.15 mg/g DW, which was 1.48 times that of HA and 1.78 times that of VM, respectively. Collectively, these results indicate that the total saponins and flavonoids contents of *P. odoratum* powder were better preserved by the VMD combined with extrusion-cooking method than by the other processing methods. Besides, previous studies had found that the antioxidant activity of *P. odoratum* was primarily associated with its TSC and TFC. So that the enhancing in antioxidant activity of EC may be related to its increased levels of total saponins and total flavonoids.

**Figure 3:**
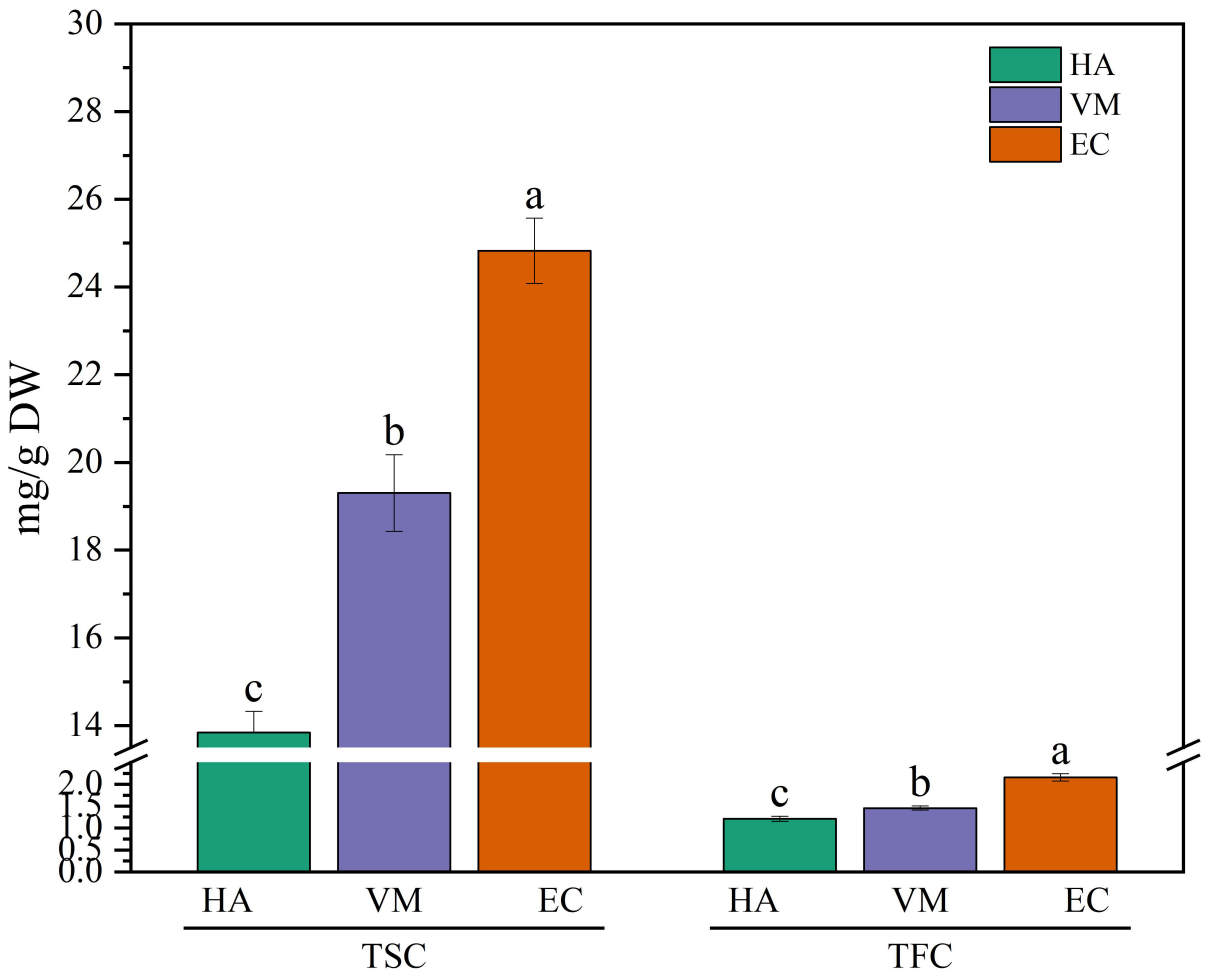
Total saponins and total flavonoids content of three samples. Error bars indicate standard deviation; different lowercase letters denote significant differences (*P* < 0.05).

### 3.4 Broad-targeted metabolomics analysis results

Broad-targeted metabolomics techniques were used to compare the three samples. As the most widely used analytical platform in metabolomics, UPLC-MS/MS has the capability to simultaneously and accurately identify and analyze compounds with a wide range of polarities and molecular weights (34). The following study then explores the microscopic-level mechanisms underlying the production of high-quality *P. odoratum* powder using the VMD combined with extrusion-cooking method.

#### 3.4.1 Metabolite profile analysis results

As shown in Figure 4A, through mass peak identification of TIC profile obtained via broad-targeted metabolomics, 333 flavonoid metabolites were detected across the three samples, including 109 flavones, 79 other flavonoids, 59 flavonols, 30 flavanones, 22 isoflavones, 17 chalcones, eight flavanols, six flavanonols, three anthocyanidins; 228 terpenoid metabolites, including 79 sesquiterpenoids, 63 monoterpenoids, 51 diterpenoids, 29 triterpenes, three triterpenoid saponins, and three terpenes; and 166 steroidal metabolites, including 145 steroidal saponins and 21 steroids.

**Figure 4:**
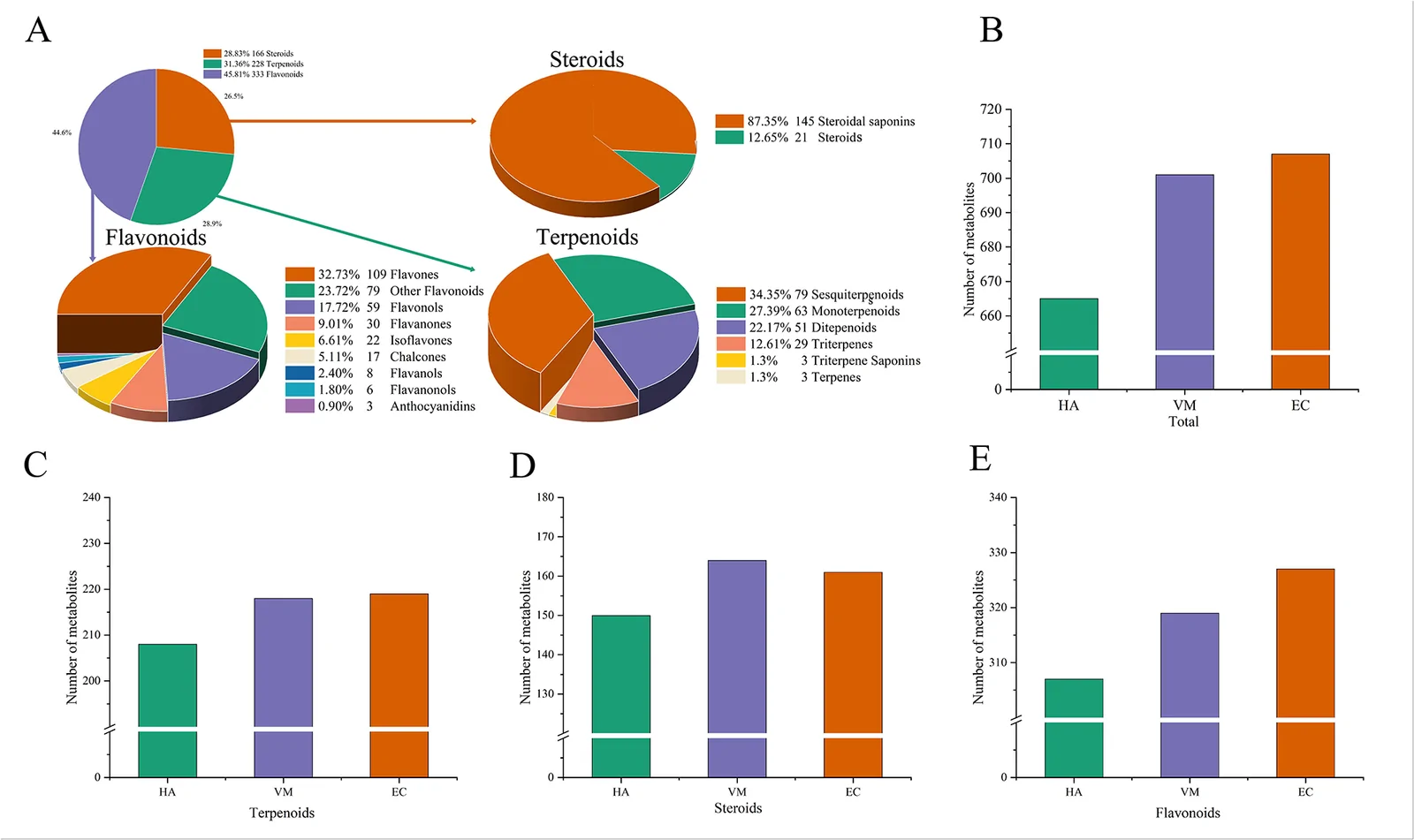
Metabolite profile analysis results. **(A)** Classification of flavonoid, terpenoid, and steroidal metabolites. **(B)** Quantitative differences in total metabolites among three samples. **(C)** Quantitative differences in flavonoid metabolites among three samples. **(D)** Quantitative differences in terpenoid metabolites among three samples. **(E)** Quantitative differences in steroid metabolites among three samples.

Based on the number of identified compounds in the three metabolite classes (flavonoids, terpenes, and steroids), EC contained the greatest number of metabolites, with 42 and 7 more than HA and VM, respectively (Figure 4B). Specifically, EC had 20 and 8 more flavonoid metabolites than HA and VM, respectively (Figure 4E), and three flavonoids were detected exclusively in EC, including tectochrysin and Epigallocatechin gallate (EGCG). Tectochrysin is a monohydroxyflavone with pronounced antioxidant and anti-inflammatory properties (35), and EGCG is a tea polyphenol that exhibits significant antibacterial, antiviral, and antioxidant activities (36). Additionally, EC contained 11 and 1 more terpenoid metabolites than HA and VM, respectively (Figure 4C), and four terpenoids were present only in EC. In contrast, VM exhibited the highest diversity of steroid metabolites, with 14 and 3 more than HA and EC, respectively (Figure 4D).

The retention of the widest variety of metabolites in EC may be attributable to the instantaneous high-temperature, high-pressure reactions occurring during extrusion-cooking, which can alter a broad range of metabolites (37). Extrusion-cooking can also promote the transformation of components by cleaving certain chemical bonds in macromolecules. For example, the hydrolysis of glycosidic bonds in flavonoid glycosides releases Eriodictyol. Additionally, the extrusion-cooking process disrupts the cell wall matrix in *P. odoratum*, thereby releasing some of the compounds bound to the cell walls. Furthermore, Maillard reactions occurring during extrusion-cooking may generate new compounds.

#### 3.4.2 Differentially analysis

PCA reduces the dimensionality of multidimensional metabolomics data to a few principal components. Thus, the differences in terms of the three metabolite types (flavonoids, terpenoids and steroids) were revealed by PCA score plot. As shown in Figure 5A, the four biological replicates of each sample formed a compact cluster, confirming that the subsequent qualitative and quantitative analyses are sufficient reproducibility and reliable. PC1 and PC2 accounted for 50.46% and 23.02% of the variance, respectively, with a cumulative contribution rate of 73.48%. HA, VM, and EC are completely separated, which indicates significant differences among the three types of *P. odoratum* powder in terms of these three metabolite categories.

**Figure 5:**
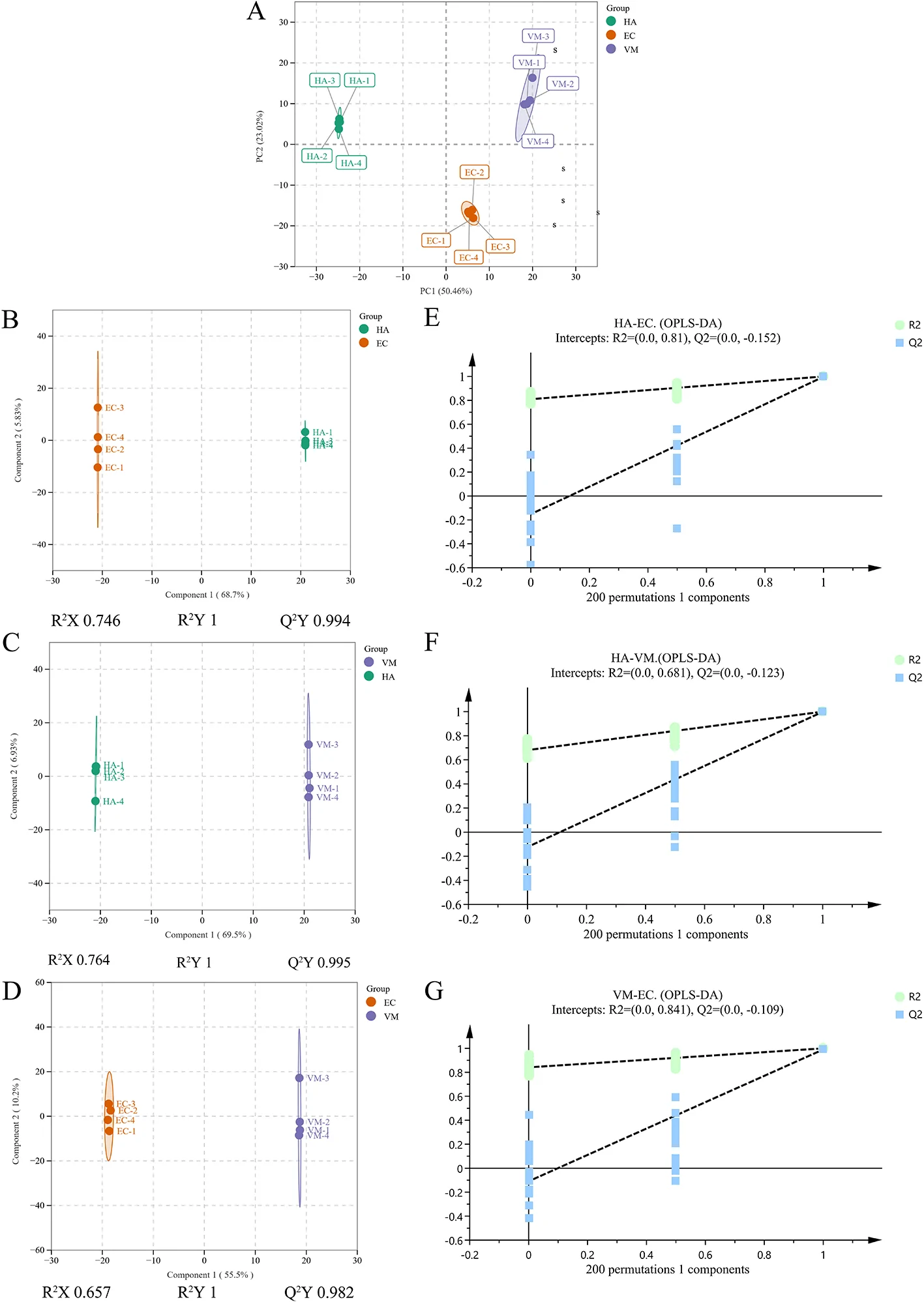
Metabolite group test analysis results. **(A)** PCA score plot of metabolites of three samples. **(B–D)** OPLS-DA model scoreplots for EC vs. HA, VM vs. HA, and EC vs. VM. **(E–G)** Permutation tests for EC vs. HA, VM vs. HA, and EC vs. VM.

To further assess the differences among the three samples, pairwise OPLS-DA models were constructed based on the three metabolite types identified by UPLC-MS/MS. The Q^2^ value, which reflects predictive ability, serves as a key parameter for evaluating model performance. When Q^2^ > 0.9 and the goodness of fit is excellent (R^2^X and R^2^Y close to 1), the model is considered reliable and stable. The OPLS-DA results were presented in Figures 5B–D, which reveal clear separations between EC and HA (*R*^2^*X* = 0.746, *R*^2^*Y* = 1, *Q*^2^ = 0.994), between VM and HA (*R*^2^*X* = 0.764, *R*^2^*Y* = 1, *Q*^2^ = 0.995), and between EC and VM (*R*^2^*X* = 0.657, *R*^2^*Y* = 1, *Q*^2^ = 0.982). All *Q*^2^ values exceeded 0.9, indicating that the models are reliable, stable, and capable of effectively explaining the metabolic variation among the three samples. In addition, permutation tests (200 permutations) were performed for the three OPLS-DA models, and the results were shown in Figures 5E–G. The *Q*^2^ intercepts for the HA-VM, HA-EC, and VM-EC comparisons were −0.123, −0.152, and −0.109, respectively. All *Q*^2^ intercepts were negative, indicating that the observed separation among the treatment groups was not attributable to overfitting.

#### 3.4.3 Different metabolites analysis

Using |FC| ≥ 2 and VIP ≥ 1 as thresholds, pairwise comparisons of differential metabolites (flavonoids, terpenoids, and steroids) were performed among the three groups. The results are presented in volcano plots (Figures 6A–C) and bar charts (Figures 6D–I). Volcano plots show the up and down regulation of differential metabolites between each pair of groups, while bar charts display the types and numbers of differential metabolites in each comparison.

**Figure 6:**
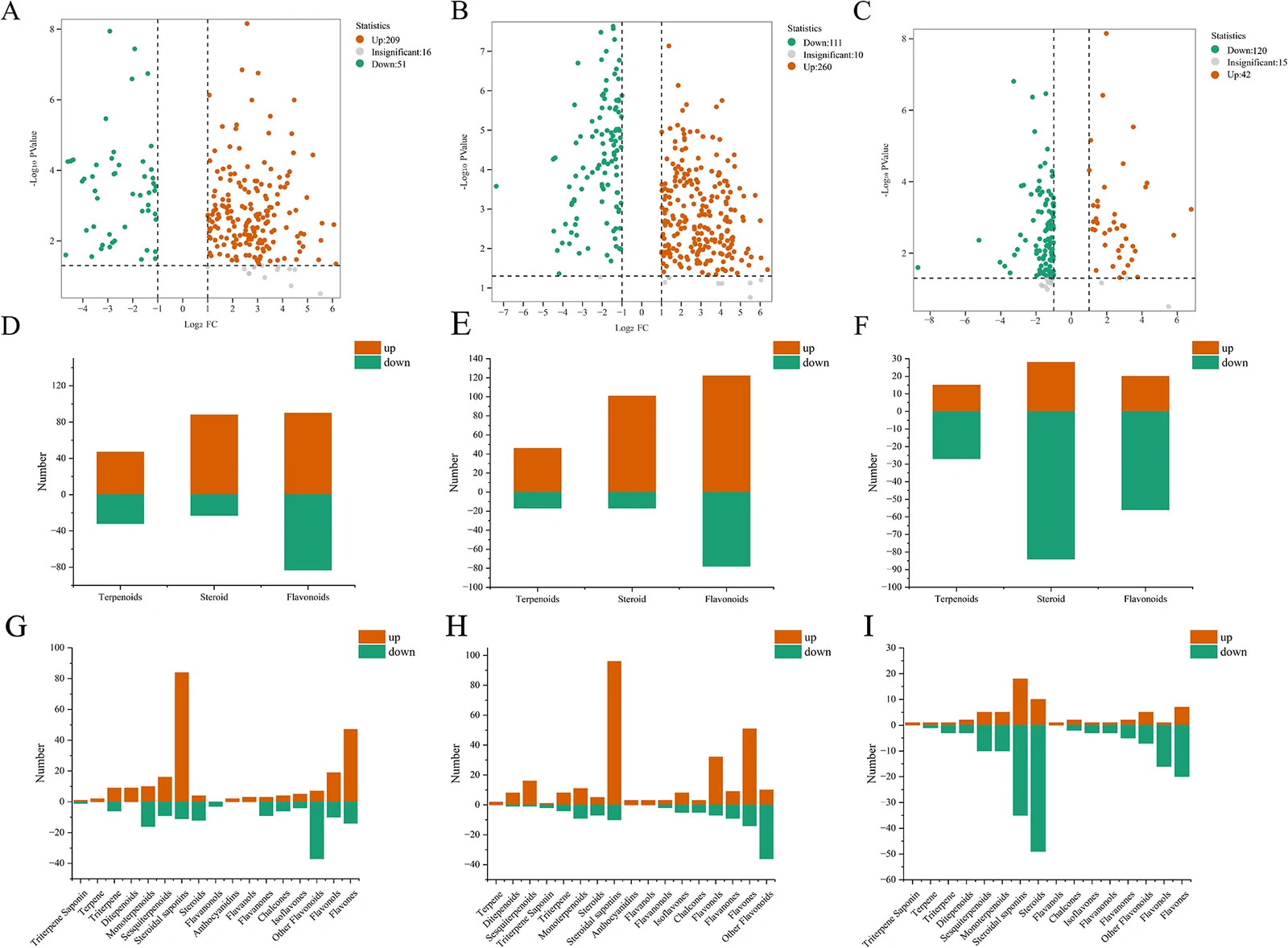
Differential metabolites between three samples. **(A–C)** Volcano plot. **(A)** EC vs. HA. **(B)** VM vs. HA. **(C)** EC vs. VM. **(D–F)** Number of differential metabolites between three samples. **(D)** EC vs. HA. **(E)** VM vs. HA. **(F)** EC vs. VM. **(G–I)** Number of differential metabolites at secondary classification levels between three samples. **(G)** EC vs. HA. **(H)** VM vs. HA. **(I)** EC vs. VM.

As shown in Figure 6A, there were 260 differential metabolites between EC and HA. Compared with HA, EC showed 209 metabolites upregulated and 51 metabolites downregulated. The specific details of different metabolites were shown in Figure 6D. Compared with HA, EC showed 90 flavonoids and 88 steroids upregulated, but 83 flavonoids downregulated. Furthermore, at the secondary classification level (Figure 6G), compared with HA, the differential metabolic in EC were primarily concentrated in three categories, including steroidal saponins, flavones and flavonols, with 84 (11), 47 (14), and 7 (37) metabolites upregulated (downregulated), respectively.

As shown in Figure 6B, there were 371 differential metabolites between VM and HA. Compared with HA, VM showed 260 metabolites upregulated and 111 metabolites downregulated. The specific details of different metabolites were shown in Figure 6E. Compared with HA, VM showed the highest number of upregulated metabolites among the flavonoids, totaling 122. At the secondary classification level (Figure 6H), compared with HA, the differential metabolic in VM were primarily concentrated in three categories, including steroidal saponins, flavones, and flavanols, with 96 (10), 51 (14), and 32 (7) metabolites upregulated (downregulated), respectively.

As shown in Figure 6C, there were162 differential metabolites between EC and VM, Compared with VM, EC showed 42 metabolites upregulated and 120 metabolites downregulated. The specific details of different metabolites were shown in Figure 6F, compared with VM, EC showed a higher number of downregulated flavonoid (38) and steroidal (84). At the secondary classification level (Figure 6I), compared with VM, the differential metabolic in EC were primarily concentrated in three categories, including steroidal saponins, saponins, and flavones, with 10 (49), 18 (35), and 7 (20) metabolites upregulated (downregulated), respectively.

Above results indicated that three processing methods primarily influenced the secondary metabolites of sterols, flavonoids, and terpenoids in *P. odoratum* powder.

#### 3.4.4 Analysis of differential metabolite enrichment

KEGG database is an effective tool for analyzing metabolic pathways and their interrelationships, enabling the visualization of diverse metabolic pathways through diagrams. Therefore, enrichment analysis of differential metabolites in three samples was contented using KEGG database to obtain comprehensive functional information, and the results were shown in Figures 7A–C. As shown in these figures, the differential metabolites among the three samples were primarily associated with pathways such as “Flavonoid biosynthesis (ko00941),” “Flavone and flavonol biosynthesis (ko00944),” “Secondary metabolite biosynthesis (ko01110),” and “Metabolism biosynthesis (ko01100)” pathways. The highest enrichment of pathway was “Flavonoid biosynthesis” (11/18, 61.11%, EC vs. HA; 11/21, 52.38%, VM vs. HA; 11/21, 52.38% EC vs. VM), followed by “Flavone and flavonol biosynthesis” pathway (4/8, 50%, EC vs. HA; 4/8, 50%, VM vs. HA; 6/12, 50%, EC vs. VM).

**Figure 7:**
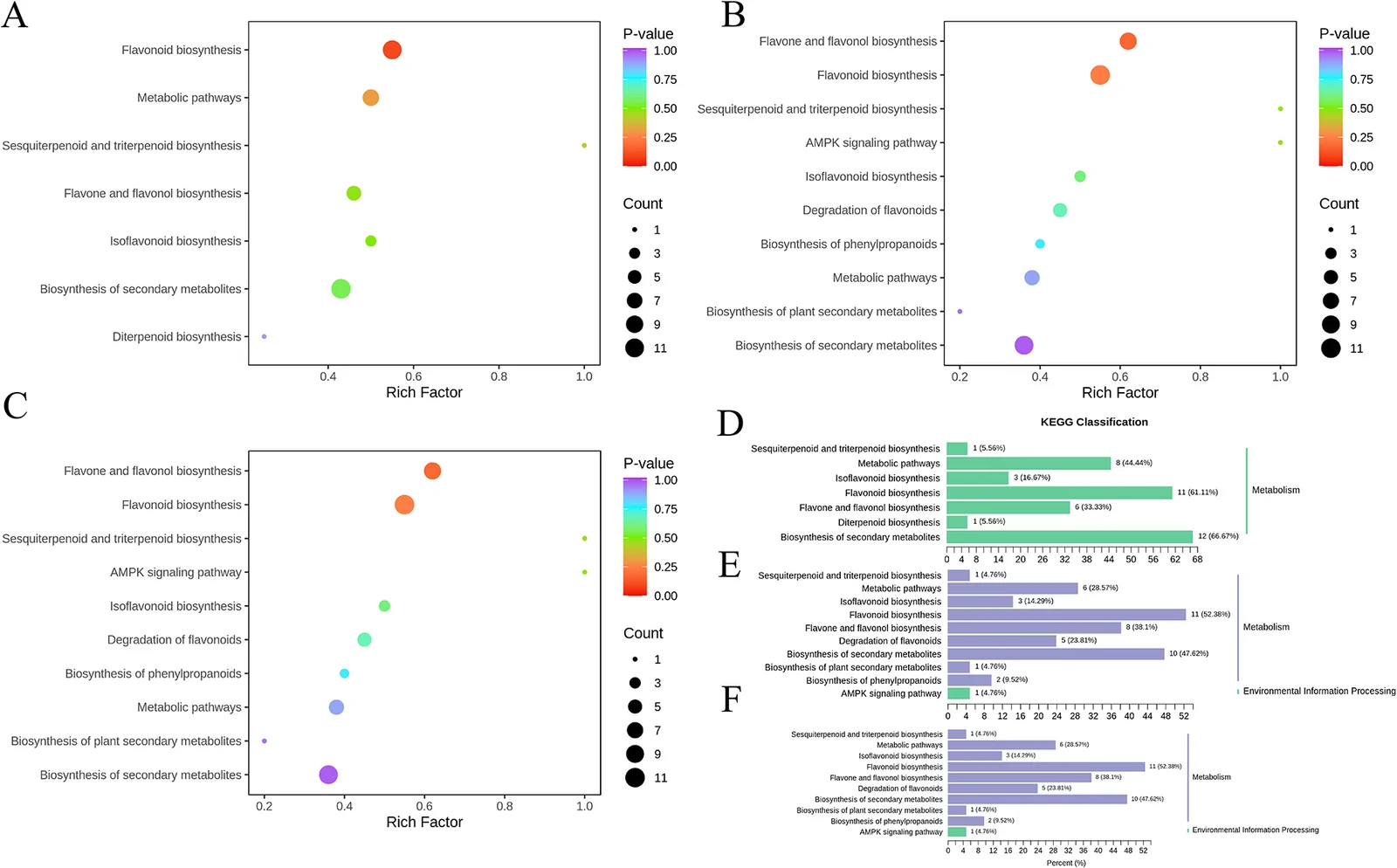
Results of differential metabolite enrichment KEGG. **(A–C)** Differential enrichment bubble plots. **(A)** EC vs. HA. **(B)** VM vs. HA. **(C)** EC vs. VM. Each bubble represents a metabolic pathway, with its horizontal coordinate and bubble size jointly indicating the magnitude of the pathway's influencing factors. Larger bubbles denote greater influence factors. Bubble color represents the P from enrichment analysis, with darker shades indicating higher enrichment levels. **(D, E)** Differential enrichment classification plots. **(D)** EC vs. HA. **(E)** VM vs. HA. **(F)** EC vs. VM.

Compared with HA, EC significantly upregulated the levels of oleuropein, apigenin, and 3,4,2′,4′,6′-pentahydroxychalcone-4′-O-glucoside in “Flavonoid biosynthesis” pathway, as well as significantly upregulated the levels of vitexin, vitexin 2″-O-β-D-glucoside, rutin, and apigenin were significantly upregulated in the “Flavone and flavonol biosynthesis” pathway (Figure 7D).

Compared with HA, VM significantly increased the levels of hesperidin, kaempferol, naringin, and epicatechin in “Flavonoid biosynthesis” pathway (Figure 7E), as well as significantly increased the levels of vitexin, vitexin 2″-O-β-D-glucoside, isovitexin, isovitexinsaponin, rutin, and apigenin in “Flavone and flavonol biosynthesis” pathway. While the VM group exhibited the greatest number of upregulated metabolites, this may contribute to the processing conditions. During HA processing, flavonoids are susceptible to oxidation due to exposure to atmospheric oxygen, resulting in the degradation of certain compounds (39). In contrast, VM processing in vacuum conditions effectively reduces oxidation reactions and facilitates the retention of these bioactive constituents (40), such as hesperetin, phloretin, and phlorizin. In addition, VM may enhance the conversion of apigenin, a key intermediate in “Flavone and flavonol biosynthesis” pathways, into downstream metabolites, thereby contributing to the increased accumulation of compounds such as vitexin and its derivatives, isovitexin and its derivatives, and cosmosin.

Compared with VM, EC significantly upregulated levels of eriodictyol, 3,4,2′,4′,6′-pentahydroxychalcone, and 3,4,2′,4′,6′-pentahydroxychalcone-4′-O-glucoside in “Flavonoid biosynthesis” pathway (Figure 7F). It is possible that the increase in eriodictyol content is due to the fact that the extrusion-cooking process broke down some of the flavonoid glycosides bound to sugars, thereby releasing the flavonoids they contained.

#### 3.4.5 Analysis of commonly differential metabolites

In this study, 78 metabolites were identified as common differentiating metabolites among EC vs. HA, VM vs. EC, and VM vs. HA (Figure 8A), which included 48 steroids, 21 flavonoids and nine terpenoids.

**Figure 8:**
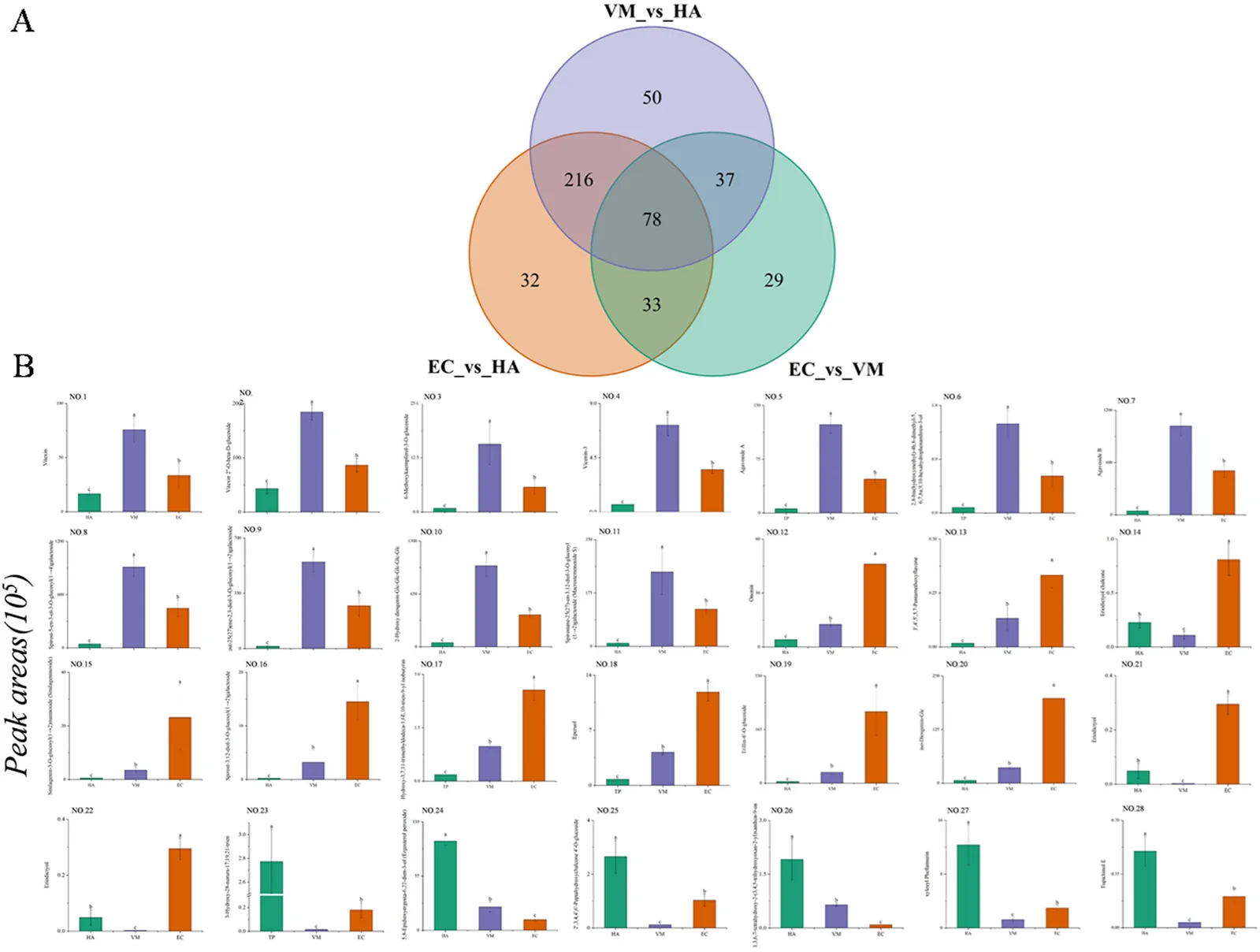
Results of commonly differential metabolites. **(A)** Venn diagram illustrating. **(B)** Peak areas of 28 key differentially expressed metabolites common to EC vs. HA, VM vs. HA, and EC vs. VM. Different letters indicate significant differences (*P* < 0.05).

Among these, 11 metabolites showed significantly higher levels in EC than in HA and VM, including five steroidal, four flavonoid and two terpenoid metabolites (Figure 8B, NO.12–22). For instance, the content of ononin (NO.12), a natural isoflavone glycoside with diverse pharmacological activities, including anti-inflammatory, antiproliferative, pro-apoptotic, and anti-metastatic effects, was 11.3-fold and 3.7 fold higher in EC than that in HA and VM, respectively (41). Similarly, the content of Eriodictyol (NO.22), a tetrahydroxyflavone widely distributed in plants and an important bioactive compound in *P. odoratum*, was 4.5 times and 82.7 times higher in EC than that in HA and VM, respectively. Previous studies have demonstrated that eriodictyol exhibits hypolipidemic, antioxidant, and antiproliferative activities (42). Therefore, the increased accumulation of these common components may contribute to the enhanced pharmacological activity on EC.

In contrast, six metabolites exhibited significantly higher levels in HA than in EC and VM, including four flavonoids, one terpenoid, and one steroid metabolite (Figure 8B NO.23–28). Notably, 61 metabolites were significantly enriched in VM compared with HA and EC, including 39 steroids, 16 flavonoids and 6 terpenoids. The top 11 most abundant metabolites in VM were presented in Figure 8B (NO.1–11). Among them, Vitexin (NO.1) showed the highest abundance, with levels 5.0 times and 2.6 times higher in VM than in HA and EC, respectively. Similarly, the contents of Vitexin 2″-O-β-D-glucoside (NO.2) and Vicenin-3 (NO.4) were 4.3-fold/2.1-fold and 11.6-fold/2.1-foldhigher in VM than in HA and EC, respectively. Extensive researches have indicated that vitexin and vicenin-3 possess neuroprotective properties and can improve memory function (43). Additionally, vitexin has been reported to exertprotective effects against hypertensive nephropathy (44) and regulate hyperglycemic responses and gut microbiota composition (45). Furthermore, other highly accumulated metabolites in VM (NO.5–11), including apigenin, quercetin, and silymarin derivatives, exhibited increases ranging from 1.2-fold to 27.0-fold compared with HA and EC.

Overall, compared with HA, EC, and VM exhibited better preservation of commonly differential metabolites. Since EC was produced from VM through extrusion-cooking, these results indicate that the high-shear forces generated during extrusion-cooking may enhance the release and extractability of certain metabolites, whereas heat-sensitive compounds may undergo degradation under the high-temperature and high-pressure conditions of extrusion-cooking processing. These findings are consistent with previous observations reported by Zhang et al. (17).

### 3.5 Correlation analysis

To further investigate whether changes in differential metabolites influenced the physicochemical quality and antioxidant activity of the samples, and to elucidate the underlying mechanism by which the VMD combined with extrusion-cooking method improved the quality of *P. odoratum* powder, correlation analyses were conducted between the results of differential metabolite analysis and those of color, taste, and antioxidant activity.

#### 3.5.1 Correlation between color and metabolites changes

According to the color results of this study, compared with VM and HA, the $E_{ab}^{*}$ of EC was enhanced, L^*^ value of EC was decreased. The findings on the correlation between color characteristics and metabolite changes help elucidate how metabolic alterations influence the color characteristics of *P. odoratum* powder. Therefore, the relationship between color characteristics and differential metabolites in the three samples was investigated using Spearman correlation analysis (|*r*| > 0.9, *P* < 0.05), the results are shown in Figure 9A, and detailed correlation results and the corresponding substance IDs are presented in Supplementary Table S1. It showed that 75 metabolites were related to taste in three samples, mainly flavonoid metabolites (46), followed by terpenes (13) and steroid metabolites (8). These results indicate that the color characteristics of the samples are mainly related to the content of flavonoids metabolites. And studies have shown flavonoids are the primary colorants in most plants (47).

**Figure 9:**
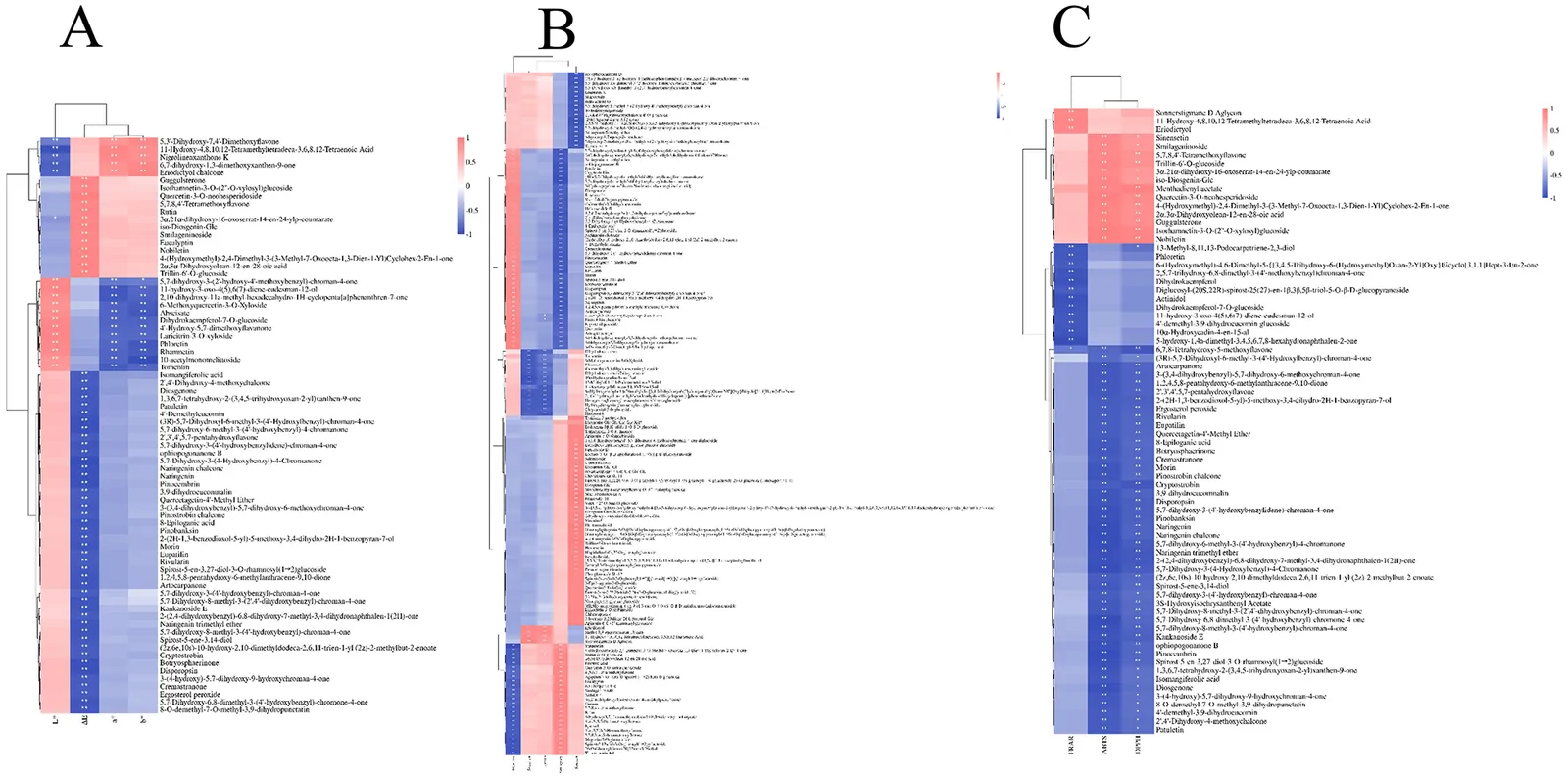
Spearman correlation analysis between physicochemical quality and differential metabolites of three samples. **(A)** Spearman correlation analysis between color and differential metabolites. **(B)** Spearman correlation analysis between taste and differential metabolites. **(C)** Spearman correlation analysis between *in vitro* antioxidant activity and differential metabolites (* indicates *P* < 0.05, ** indicates *P* < 0.01).

Specifically, 12 metabolites showed significant positive correlations with L^*^ value, including eight flavonoid metabolites and four terpenoid metabolites. Such as Phloretin, a flavonoid, studies have indicated that the presence of Phloretin inhibited the occurrence of Maillard reaction, thereby significantly reducing the formation of brown compounds produced by this reaction and enhancing the brightness of the samples (21). In this study, Maillard reaction commonly occurred during extrusion-cooking (48). However, the reduced levels of Phloretin in EC diminished their inhibitory effect on the Maillard reaction, resulting in decreased brightness of the *P. odoratum* powder processed by VMD combined with extrusion-cooking. 6 metabolites showed significant negative correlations with L^*^ value, including four flavonoid metabolites and two terpenoid metabolites. For example, correlation analysis revealed a significant negative correlation between Eriodictyol chalcone (flavonoid) chalcone and L^*^, indicating that higher Eriodictyol chalcone content results in darker powder. Schmitzer et al. (49) observed that the reduction in rose color intensity was significantly correlated with the newly formed Eriodictyol chalcone in the petals, indicating that its presence adversely affected sample brightness. This is consistent with the findings of the present study, in which EC exhibited an increase in Eriodictyol chalcone content and a decrease in the brightness of *P. odoratum*.

Five metabolites showed significant positive correlations with a^*^ and b^*^ value, including four flavonoid metabolites and one terpenoid metabolite. Twelve metabolites showed significant negative correlations with a^*^ and b^*^ value, including eight flavonoid metabolites and 4 terpenoid metabolites. Interestingly, metabolites associated with a^*^ and b^*^ values are identical to those associated with L^*^ value, but their correlations are reversed. Such as Eriodictyol chalcone, 5,3′-dihydroxy-7,4′-dimethoxyflavone, 6,7-dihydroxy-1,3-dimethoxyxanthen-9-one and Nigrolineaxanthone *K*, these four metabolites are flavonoids, all containing long conjugated systems that can absorb violet-blue light in visible light and reflect yellow light, so they are all yellow pigments. Therefore, the higher their content, the higher the b^*^ value and the lower the L^*^ value of the sample.

In this study, compared with the other two samples, the darker, scorched-yellow color of EC is primarily associated with the changes in flavonoid metabolites, especially associated with decreased levels of Phloretin, and increased levels of Eriodictiol chalcone, 5,3′-Dihydroxy-7,4′-dimethoxyflavone, 6,7-dihydroxy-1,3-dimethoxyxanthen-9-one and Nigrolineaxanthone *K*.

#### 3.5.2 Correlation between taste characteristics and metabolites changes

According to the sensory evaluation results of this study, VMD effectively reduced the acidity of *P. odoratum* powder. Compared with VM and HA, the bitterness of EC was decreased, the sweetness and astringency of EC was enhanced. The findings on the correlation between taste characteristics and metabolite changes help elucidate how metabolic alterations influence the taste characteristics of *P. odoratum* powder. Therefore, the relationship between taste characteristics and differential metabolites in the three samples was investigated using Spearman correlation analysis (|*r*| > 0.9, *P* < 0.05), the results were presented in Figure 9B, and detailed correlation results and the corresponding substance IDs are presented in Supplementary Table S2. The results showed that 153 metabolites were associated with taste in the three samples; among these, flavonoid metabolites were predominant (80), followed by steroidal metabolites (37), of which 34 were steroidal saponins. This indicates that the taste characteristics of the samples are mainly related to the content of flavonoid metabolites. And studies have shown a strong correlation between flavonoid content and taste characteristics (50). However, steroid saponins are mainly associated with the bitterness of samples, especially spirosterane-type.

Specifically, 45 metabolites showed significant positive correlations with bitterness, including 33 flavonoid metabolites, five steroidal metabolites, and seven terpenoid metabolites. Twenty-five metabolites showed significant negatively correlations with bitterness, including 13 flavonoid metabolites, six steroidal metabolites, and six terpenoid metabolites. For example, the correlation analysis revealed that the three saponin metabolites, including Ruscogenin, Spirost-5-en-3,27-diol-3-O-rhamnosyl (1 → 2) glucoside and (25R)-Spirost-4-ene-3,12-dione (steroid saponin), all showed significant positive correlations with bitterness. All three metabolites are spirostanol-type steroids. Studies have shown that spirosterol-type aglycones and saponins are often described as having a bitter taste (54), so reducing the content of these three metabolites helps reduce bitterness. In this study, compared to HA and VM, the content of these three metabolites in EC was significantly reduced, speculating that the decreased bitterness of the *P. odoratum* powder produced by VMD combined with extrusion-cooking was associated with decrease in contents of Ruscogenin, Spirost-5-en-3,27-diol-3-O-rhamnosyl (1 → 2) glucoside and (25R)-Spirost-4-ene-3,12-dione.

Four metabolites showed significant positive correlations with sweetness, including one flavonoid metabolite (Eriodictyol) and three terpenoid metabolites. Although Eriodictyol itself is not intensely sweet, it has been well-documented as a potent bitter-masking flavanone that can indirectly enhance sweet taste perception, and its structural analogs have been shown to directly potentiate sweetness (51). In this study, Eriodictyol content of EC is higher than that of HA and VM, and EC's sweetness is the highest, indicating that the increased sweetness of the *P. odoratum* powder produced by VMD combined with extrusion-cooking was associated with an increase in Eriodictyol content. Fourteen metabolites showed significant negatively correlations with sweetness, including seven flavonoid metabolites, two steroidal metabolites, and five terpenoid metabolites. For example, the correlation analysis revealed that Rhamnetin (flavonoid) showed a significant negative correlation with sweetness. Previous studies had indicated that Rhamnetin is a bitter compound (9), indicating that lower Rhamnetin content results in higher sweetness in the tea infusion. In this study, EC exhibited a lowest Rhamnetin content and the highest sweetness, indicating that the increased sweetness of the *P. odoratum* powder produced by VMD combined with extrusion-cooking was associated with a decrease in Rhamnetin content.

Twenty-six metabolites showed significant positive correlations with astringency, including 13 flavonoid metabolites seven steroidal metabolites, and 6 terpenoid metabolites. Forty-six metabolites showed significant negatively correlations with astringency, including 34 flavonoid metabolites, five steroidal metabolites, and seven terpenoid metabolites. Research have shown that astringency is mainly produced by polyphenolic compounds (such as tannins) binding and precipitating with salivary proteins. Flavonoid metabolites, a type of polyphenol, contain galloyl groups, catechin structural units, and free ortho-dihydroxyl groups, all of which are closely related to astringency (52). For example, Rutin and Quercetin-3-O-neohesperidoside showed significant positive correlations with astringency, indicating that the higher contents of Rutin and Quercetin-3-O-neohesperidoside, the higher astringency. In this study, compared with HA and VM, the astringency of EC was the highest, and the TFC and the contents of Rutin and Quercetin-3-O-neohesperidoside were highest, suggesting that the increased astringency of the *P. odoratum* powder produced by VMD combined with extrusion-cooking was associated with an increase in total flavonoids content, such as Rutin and Quercetin-3-O-neohesperidoside. Besides, 3α,21α-dihydroxy-16-oxoserrat-14-en-24-ylp-coumarate (terpenoid) contains a para-coumarate structural unit. And the para-coumaric acid and its derivatives are typical phenolic acids, with a certain astringency and astringent properties (46). In this study, 3α,21α-dihydroxy-16-oxoserrat-14-en-24-ylp-coumarate showed significant positive correlations with astringency, and the content of 3α,21α-dihydroxy-16-oxoserrat-14-en-24-ylp-coumarate in EC is higher than that in HA and VM, indicating that the increased astringency of the *P. odoratum* powder produced by VMD combined with extrusion-cooking was associated with an increase in content of 3α,21α-dihydroxy-16-oxoserrat-14-en-24-ylp-coumarate.

Compared with VM, EC exhibited reduced bitterness and increased sweetness and astringency. This is due to the high-temperature, high-pressure conditions in the extrusion-cooking process, which change the total flavonoid content and total saponins content, especially resulting in reduced bitter compounds (such as Ruscogenin) and increased sweet compounds (such as Eriodictyol) and astringent compounds (such as Rutin). Notably, although astringency of *P. odoratum* powder tea infusion increased after the extrusion-cooking process, astringency plays a crucial role in the sensory experience of the tea infusion. Furthermore, astringency is a key factor influencing the sensory characteristics of food, tea, and other beverages. This improvement made *P. odoratum* powder infusion more complex and palatable, thereby increasing the appeal of *P. odoratum* powder processed by extrusion-cooking.

#### 3.5.3 Correlation between antioxidant activity and metabolites changes

The antioxidant activity results obtained in this study were consistent with the changes in functional component contents. To further investigate the correlation between the antioxidant activity of *P. odoratum* powder and its differential metabolites, a Spearman correlation analysis was performed (|*r*| > 0.9, *P* < 0.05). Detailed correlation results and the corresponding substance IDs are presented in Supplementary Table S3. As shown in Figure 9C, 74 metabolites were found to be significantly correlated with antioxidant activity, mainly flavonoid metabolites (48), followed by terpenes (18) and steroid metabolites (9). These results indicate that the antioxidant activity of *P. odoratum* powder was significantly correlated with flavonoid metabolites, whereas only a small number of steroid and terpenoid metabolites showed a significant positive correlation, indicating that changes in antioxidant activity are associated with flavonoid metabolites. This is consistent with the findings of most studies on the antioxidant activity of *P. odoratum* (11), the antioxidant activity of *P. odoratum* powder is primarily associated with its flavonoids. This is mainly related to the predominance of phenolic hydroxyl groups in the structures of flavonoid metabolites, while phenolic hydroxyl groups are not commonly present in steroid and terpene metabolites (6).

Specifically, three metabolites exhibited significant positive correlations with the FRAP reducing capacity, including one flavonoid metabolite (Eriodictyol) and three terpenoid metabolites. Researches had found that Eriodictyol, one of the main flavonoids in juice, had a significant iron-reducing ability and contributes a significant portion of its total antioxidant activity, indicating that the higher content of Eriodictyol, the higher of iron-reducing ability (53). According to the results of LC-MS, the content of Eriodictyol in EC was higher than that in HA and VM, suggesting that the enhanced antioxidant activity of *P. odoratum* powder produced by VMD combined with extrusion-cooking was associated with an increase in the level of Eriodictyol. Thirteen metabolites showed significant negative correlations, including 6 flavonoid metabolites, six steroidal metabolites and one terpenoid metabolite.

Thirteen metabolites showed significant positive correlations with the ABTS^+^• and DPPH• scavenging rate, including five flavonoid metabolites, four steroidal metabolites and four terpenoid metabolite. Forty-six metabolites exhibited significant negative correlations with the ABTS^+^• and DPPH• scavenging rate, including 36 flavonoid metabolites, four steroidal metabolites and six terpenoid metabolite. Interestingly, metabolites associated with ABTS^+^• scavenging rate and DPPH• scavenging rate are highly similar, only 13-Methyl-8,11,13-Podocarpatriene-2,3-diol showed significant negative correlations with the DPPH• scavenging rate. Structural research has found that Quercetin-3-O-neohesperidoside is the most classic flavonol antioxidant because of the orthodiphenol hydroxyl structure, and the methoxy group in Isorhamnetin-3-O-(2″-O-xylosyl) glucoside can enhance the antioxidant activity of the adjacent hydroxyl group through electronic effects (6). The higher the flavonoid content of these two flavonoids, the stronger the antioxidant activity of the sample. In this study, these two flavonoid contents of EC are higher than that of HA and VM, indicating that the enhanced antioxidant activity of *P. odoratum* powder produced by VMD combined with extrusion-cooking was associated with an increase in the level of Quercetin-3-O-neohesperidoside and Isorhamnetin-3-O-(2″-O-xylosyl) glucoside. Besides, the antioxidant activity of EGCG (only found in EC) is at least 100 times greater than that of vitamin C and 25 times greater than that of vitamin E (38). This may be one of the key reasons why EC demonstrated stronger antioxidant activity than HA and VM.

In summary, the significant enhancement of antioxidant activity in EC is primarily associated with the increased content of flavonoids, such as EGCG, Eriodictyol, Quercetin-3-O-neohesperidoside, and so on. Additionally, reactions occurring during the extrusion-cooking process may also affect other components, and further investigation is needed to assess their impact on composition and antioxidant activity. Furthermore, the Maillard reaction that occurs during extrusion-cooking generates certain compounds with strong antioxidant activity (48). Therefore, it is suggested that *P. odoratum* powder processed by VMD combined with extrusion-cooking holds potential for food industry applications, as it may amplify the beneficial health effects of *P. odoratum* tea infusions.

## 4 Conclusion

This study found that compared with *P. odoratum* powder produced using hot-air drying or vacuum microwave drying (VMD), the powder produced using VMD combined with extrusion-cooking (EC) exhibited significant improvements in both physical quality and antioxidant activity. This is closely related to the ability of this process to better preserve flavonoid metabolites in *P. odoratum*. These improvements are primarily manifested as follows: EC exhibited a more yellowish color, which was mainly associated with reduced levels of Phloretin and increased levels of Eriodictyol chalcone; bitterness was significantly reduced, with more pronounced sweetness and astringency, primarily due to lower levels of Ruscogenin (a bitter compound) and higher levels of Eriodictyol (a sweet compound) and Rutin (an astringent compound); higher antioxidant activity was primarily associated with increased levels of Eriodictyol and Quercetin-3-O-neohesperidoside, as well as the emergence of EGCG. Notably, the more yellowish color of EC, enhanced sweetness and astringency, and increased antioxidant activity were all associated with high contents of flavonoid metabolites, whereas the decrease in bitterness was associated with low contents of steroidal saponins. Overall, EC produces a more visually appealing color, a richer and more palatable tea infusion, and stronger antioxidant activity, thereby offering greater application potential in the food industry. However, it is worth noting that while EC generates new metabolites, the extrusion-cooking process can significantly degrade certain heat-sensitive components, thus reducing their content. In practice, different processing methods should be selected for *P. odoratum* based on the specific application scenario. These findings provide new insights into the selection of processing methods for *P. odoratum*. Future research should focus on gaining a deeper understanding of how processing affects the active components and efficacy of *P. odoratum*, as well as the potential mechanisms underlying these effects.

Besides, compared with hot-air drying and VMD process, the VMD combined with extrusion-cooking process integrates the advantages of VMD, including rapid moisture removal and efficient drying, with those of extrusion cooking. During extrusion cooking, the combined effects of high temperature, pressure, and shear forces promote starch gelatinization, protein denaturation, and the inactivation of enzymes and microorganisms, while simultaneously modifying the structural characteristics and water distribution of the material, thereby contributing to improved storage stability and extended shelf life. Therefore, this integrated process exhibits considerable potential for continuous and large-scale processing of root and tuber crops. However, the scale-up of extrusion cooking requires precise control of critical processing parameters, including barrel temperature, screw speed, and feed moisture content, to ensure consistent product quality, as excessive local heating may accelerate the degradation of heat-sensitive bioactive compounds such as saponins. Moreover, the initial capital investment and maintenance costs associated with twin-screw extruders should be carefully balanced against the potential economic benefits arising from reduced downstream milling energy consumption, enhanced processing efficiency, and improved product quality. Future studies should focus on comprehensive techno-economic assessments, pilot-scale validation, and shelf-life evaluations to further demonstrate the feasibility of adopting extrusion cooking as a key unit operation for the industrial processing of *P. odoratum*.

## Data Availability

The original contributions presented in the study are included in the article/Supplementary material, further inquiries can be directed to the corresponding authors.
